# Entangled Vulnerabilities: Gendered and Racialised Bodies and Borders in EU External Border Security

**DOI:** 10.1080/14650045.2023.2291060

**Published:** 2024-01-04

**Authors:** Julia Sachseder, Saskia Stachowitsch, Madita Standke-Erdmann

**Affiliations:** a Department of International Relations and Department of Gender Studies, Central European University, Vienna, Austria; b Department of War Studies, King’s College London, London, UK

## Abstract

The notion of vulnerability is gaining traction in EU border protection. On the one hand, the concept refers to vulnerable migrants and their affectedness by insecurity and violence. On the other, it indicates the susceptibility of borders to irregular crossings and cross-border crime. Both forms of vulnerability are assessed through dedicated procedures under the umbrella of the European Border and Coast Guard Agency Frontex. To make sense of these seemingly contradictory conceptual and practical uses of vulnerability, we draw on feminist postcolonial scholarship in security studies and political geography. We argue that a shared colonial matrix of gendered and racialised meanings and problematisations enables analogies between borders and bodies as (un-)deserving of protection. In a discourse-theoretical analysis of Frontex documents, we show how the ambiguous use of vulnerability legitimises the EU border regime and its security practices by constructing EU bordering as neutral and objective and EU borders as objects of care. We conclude that vulnerability becomes increasingly important for normalising the EU’s violent borders in the context of the EU’s broader claims to liberal values of freedom, protection, and human rights.

## Introduction

The external borders of the EU have turned into the world’s deadliest and most violent border regions (Albahari 2015; Horsti 2019). Despite the EU’s continued claim to liberal values of freedom and protection, human rights violations have become rampant, including illegal push-backs that are tolerated or even conducted by the central EU border security actor, the European Border and Coast Guard Agency Frontex (Christides and Lüdke 2022; correctiv 2019; Davies, Isakjee, and Obradovic-Wochnik 2022; Tondo 2021). Additionally, human rights and the protection of migrants and refugees are being hollowed out in legal and policy frameworks, as evident in the most recent reform of the Common European Asylum System (CEAS) (Council of the European Union 2023). The EU thus produces insecurity and violence through progressively securitised borders (Fontana 2021) and migration policies (Léonard and Kaunert 2020). This process is gendered and racialised, i.e. it affects migrant women and marginalised groups disproportionately, is often legitimised on the basis of gendered and racialised stereotypes and binaries (Freedman 2016; Gerard 2016; Gerard and Pickering 2014; Pickering and Cochrane 2013), and reproduces global hierarchies rooted in colonial power relations (Moffette and Vadasaria 2016).

One way through which the EU attempts to mitigate the contradictions and tensions between its liberal values and border violence is the concept of vulnerability, based upon which authorities like Frontex claim to organise their mandate of protection and humanitarian care at the outskirts of the EU’s border regime. This concept has been institutionalised in EU border management with the implementation of the so-called EU-Turkey Deal in 2016 as an assessment, measuring, and screening tool for migrants’ deservingness of protection (Papada 2023; Tazzioli 2018; Welfens and Bekyol 2021). International humanitarian organisations, like the UNHCR, and EU-member states have integrated these vulnerability assessments into their asylum procedures (Welfens 2023) and they are currently also being rolled out under the EU’s framework of Integrated Border Management (IBM)^1^ for which Frontex is largely responsible. An increased focus on vulnerability is supposed to identify those with a heightened need for protection and ensure appropriate treatment (Freedman 2019). However, the criteria for vulnerability and associated eligibility for protection are continuously narrowed down, e.g., to medical conditions, and perpetuate problematic gendered and racialised stereotypes. These include assuming women to be automatically vulnerable (Freedman 2019) or marking racialised groups as less deserving (Moffette and Vadasaria 2016; Turner 2019).

At the same time, the EU has introduced another, quite different understanding of vulnerability, expressed in the idea of vulnerable borders. This idea manifests in specific and politically consequential knowledge practices that are also called ‘vulnerability assessments’ (Fjørtoft 2020) which measure the risk of illegal and irregular cross-border activities. In this context, vulnerability refers to the permeability of borders, territories, and infrastructures and their susceptibility to unwanted border crossings, cross-border crime, smuggling, and document fraud, and the security threats that may result from such activities. For the EU external borders, standardised assessments of vulnerability are conducted as part of Frontex’s risk analysis, which evaluates risks to borders on the basis of extensive data collection and surveillance, and thereby determines when, where, and how Frontex operations are conducted (Horii 2016; Horsti 2012; Neal 2009; Paul 2017). These assessments provide the basis for intervention into member states’ border protection, including the deployment of Frontex units to their territory, allocation of resources to high-risk border sections, and justification of increased power transferral to Frontex (Paul 2017; see also Rijmpaa 2016, 14).

This paper takes these seemingly contradictory uses of vulnerability – as a humanitarian concept on the one hand, and an instrument of border securitisation on the other – as an analytical starting point for investigating the EU’s liberal border governance. Thereby, it scrutinises the inherent contradiction in this form of governance which is guided by a universalist normative framework centring freedom of choice and movement, while ‘deploy[ing] force and violence’ (Dillon and Reid 2001, 41) to guarantee control over bodies on the move. From a feminist postcolonial perspective, we argue that a shared colonial matrix underpins both concepts and assessment practices and connects them through gendered and racialised meanings and problematisations. By studying how both migrants and borders are discursively produced as vulnerable and how these different discourses – both institutionalised under the IBM approach and Frontex – inform each other, we investigate vulnerability’s growing importance for normalising violent borders. We show that, similar to migrant vulnerability, the notion of border vulnerability relies on intersectionally gendered and racialised hierarchies, dichotomies, and normative judgements that are constituted by coloniality. Vulnerability not only enables the strategic inclusion and exclusion of migrants in/from protection by ascribing or denying the status of being vulnerable (Papada 2023; Turner 2019). It also allows for revaluing borders as objects of humanitarian care, rather than simply securitised protection, that are prioritised over the security of people on the move. This manifests, for example, in portrayals of EU borders as feminised, vulnerable spaces to be protected from dangerous masculinised and racialised Others. These constructions enable analogies between people and spaces as (un-)worthy and (un-)deserving of protection and care (Shilliam 2018; Welfens 2023). This, in turn, bridges over the perceived contradiction between the EU’s self-ascribed liberal values on the one hand, and border violence on the other.

We present our argument as follows: We first critically engage with the literature on the ambiguous normative assumptions and meanings of vulnerability with regards to migrants/refugees, and the role of gender, race, and coloniality therein. We identify a lack in conceptual and empirical work on the gendered and racialised constructions of vulnerable borders and how they interact with notions of migrant vulnerability. We draw on feminist and postcolonial approaches in security studies (Adamson 2020; Barkawi and Laffey 2006; Howell and Richter-Montpetit 2019; Sjoberg 2010; Tickner 1992; Wibben 2011) and political geography (Dixon 2016; Hyndman 2019; Massaro and Williams 2013; Pratt 1999, 2005) to develop a discourse-theoretical approach (Doty 1996; Shepherd 2008) to border vulnerability as a gendered and racialised concept that is co-constitutive of migrant vulnerability. With this framework, we examine how Frontex conceptualises and uses vulnerability in its training material for border guards, risk analysis reports, and strategy, programming, and governance documents, ranging from 2013 to 2022. We study Frontex as a key actor in conceptualising and implementing both variants of vulnerability in the name of protecting European states and the so-called liberal values that are constitutive of the ‘European idea’ (Isakjee et al. 2020, 1754), while also being deeply implicated in violent border practices (Glouftsios 2023; Perkowski 2021).

Our discourse-theoretical analysis elucidates that constructions of border and migrant vulnerability are linked to each other through discursive processes of gendering and racialisation expressed in the feminisation of borders as the object of protection and care and the racialisation of the non-EU Other as threat. This enables the construction of the EU as a white and masculine security actor (Sachseder and Stachowitsch 2023), feeding into two key discourses that underpin liberal governance: humanitarianism (Reid‐Henry 2014; Rutazibwa 2019) and neutral knowledge production (Bruff and Tansel 2019; Shilliam 2016). Together, both understandings of vulnerability thereby obscure structural and intersecting root causes of individual harm-ability, whilst leaving normative claims to EUropean^2^ values as linked to rights, freedom, and security intact. Reading the different conceptualisations and practices of vulnerability together in the institutional context of Frontex from a feminist postcolonial perspective thus provides key insights into the multiple ways in which vulnerability generates legitimacy for problematic and violent border practices.

## Vulnerability, Migration, and (EU) Border Security

Vulnerability is a contested concept (Browne, Danely, and Rosenow 2021; Cole 2016; Cooper 2015; Heath-Kelly and Gruber 2023; Virokannas, Liuski, and Kuronen 2018) with diverse trajectories ranging from feminist moral philosophy and an ethics of care (Butler 2009; Butler, Gambetti, and Sabsay 2016; Fineman 2008, 2017; Mackenzie, Rogers, and Dodds 2013) as well as humanitarian discourses (Daoust and Dyvik 2021; Turner 2019) to a focus on the condition and resilience of state security, territory, infrastructures, and weapons systems (Cohn 2014; for a concise genealogy see; Lorenz 2018). As such, the meaning of vulnerability is not stable, but amenable to diverse managerial, militarised, or humanitarian discourses and practices which can, in principle, produce both emancipatory effects and spaces for inclusion as well as exclusions and insecurities. While migration and border studies have largely focused on embodied, humanitarian, and subject-related perspectives on vulnerability (Reilly, Bjørnholt, and Tastsoglou 2022), less attention has been paid to the militarised and securitised version of the concept concerned with risks to and permeability of borders that is gaining traction in EU border security (Fjørtoft 2020). Importantly, how gender and race matter in the concept of border vulnerability, how it is co-constitutive with the gendered and racialised concept of migrant vulnerability and how both are structured by and connected through coloniality has so far not been systematically studied.

In migration and border studies, vulnerability has mainly been understood as an indicator that points to the spatial and temporal expressions and consequences of multiple insecurities that migrants encounter in transit and destination countries (Fontana 2021; Rasuly-Paleczek 2020; Stewart and Mulvey 2014). While some studies have focused on how vulnerable migrants can be identified more effectively during reception and return procedures (Heuser, Junghans, and Kluth 2021), others have used the concept to problematise various bordering practices that create constantly changing forms of (in)security of spaces and bodies (Fontana 2021). This latter perspective challenges the emancipatory potential of vulnerability in broader humanitarian frameworks (Howden and Kodalak 2018; Janmyr and Mourad 2018; Johnson 2011; Olivius 2016; Ticktin 2011). As Sözer (2020) demonstrates, for example, vulnerability is an essential category within neoliberal humanitarianism – a product and symptom of global political and economic transformation into neoliberal government. As a key tool to assess, categorise, measure, monitor, and ultimately exclude migrant bodies, it allows for cost- and time-efficient, ‘targeted’ assistance and makes selective humanitarian practice intelligible, while reducing the numbers of those eligible for support (Cantat 2018; Robinson 2022). Translated into institutions, programmes, and practices, vulnerability thus serves as an unevenly distributed exclusionary label within the (neo-)liberal politics of humanitarianism (Reid‐Henry 2014; Sözer 2020).

In doing so, vulnerability also reproduces the colonial legacies of humanitarianism (Skinner and Lester 2012), particularly in the ways in which it discursively divides the world into vulnerable and non-vulnerable territories and populations (Bankoff 2001). As such, vulnerability ‘belong[s] to a knowledge system formed from within a dominant Western liberal consciousness’ (Bankoff 2001, 29) that continuously reproduces essentialist notions of the ‘vulnerables of the South’, serving to ‘uphold global power relations’ of inequality (Lorenz 2018, 68; see also Cole 2016). Colonial notions of gender and race are central to sustaining dichotomies between (non-)vulnerable subjects (Turner 2019). As such, racialised women are often foregrounded as inherently vulnerable and/or interventions are justified ‘in the name of gender equality’ (Olivius 2016, 272). In turn, the suffering of LGBTIQ* or cis-male refugees are largely silenced (Bird 2022).

The gendered and racialised politics of vulnerability is reflected in the highly selective procedures currently established in the EU border regime to assess migrants’ vulnerability (FitzGerald 2012), which facilitate faster access to the EU’s asylum system for some (Freedman 2019), but criminalise and illegalise an increasing number of others. At the EU external borders, gendered expectations, patriarchal family norms, and willingness to assimilate to a rather rigid set of national and EUropean norms and values (Welfens 2023) provide the basis for assessing vulnerability and deservingness (Welfens and Bonjour 2020). This fosters stereotypes of masculinised migrants as threatening, and feminised migrants as passive and agency-less, and racialises them by weighing harm-ability against perceived threats and security risks emanating from the Other (Tazzioli 2018, 2021). As part of humanitarian bordering (Pallister-Wilkins 2015; Perkowski 2018), vulnerability thus sustains and legitimises violent and repressive border regimes on the basis of white supremacy (Pallister-Wilkins 2021), patriarchy, and the paradoxical reproduction of migrants as ‘a risk’ and ‘at risk’ (Aradau 2004).

Taken together, critical scholarship on migration and vulnerability has thus shown how conceptualisations of migrants as vulnerable draw on and reproduce gendered and racialised stereotypes, binaries, and hierarchies. Less scholarly attention has been paid to how vulnerability contributes to the upholding of power relations when linked to territories, borders, and security infrastructure – often in ways that invisibilise human vulnerability (Cohn 2014). This is despite scholarship that has shown how the notion of vulnerable territories is rooted in colonial ‘imaginative geographies’ (Said 2003, 49) and linked to vulnerable populations in ways that divide the world into more and less dangerous/unsafe/vulnerable spaces (Bankoff 2001; Mason 2014). However, an analysis of the ways in which gender and race, as colonial articulations, structure understandings of vulnerable territories, including borders, and link them to migrants’ vulnerability remain unstudied.

This perspective is needed as the EU is currently establishing an understanding of vulnerability as a volatile and threatening condition to which borders are constantly exposed (Bissonnette and Vallet 2021). As Fjørtoft (2020) has shown, Frontex has institutionalised such an understanding of border vulnerability in its wider practice of risk analysis. Employing the so-called Vulnerability Assessment Methodology (Frontex 2016a) that quantifies and datafies judgements on risk, these vulnerability assessments are envisioned as a depoliticised, technical, knowledge-based tool to elevate external border issues above the political preferences of member states (Fjørtoft 2020). Yet, they remain highly political in that they mitigate power struggles in the EU, legitimise challenges to member states’ sovereignty, and result in the agency’s continuously expanded and far-reaching competences. This is achieved through depoliticised claims of objectivity and quantifiability (Fjørtoft 2020, 561) within the logics of neoliberal governance that centres efficiency, (cost-)effectiveness, and allocation of scarce resources (Paul 2017). While research shows how Frontex’s risk analysis in general promotes gendered and racialised problematisations of migration and migrants (Stachowitsch and Sachseder 2019), a feminist postcolonial analysis of how gender and race inform the discursive construction of border vulnerability and adjacent assessment practices in the EU is missing. In the next section, we therefore develop a discourse-theoretical approach to border vulnerability and how it relates to migrant vulnerability through colonial hierarchies of gender and race.

## A Discourse-Theoretical Approach to the Connection between Migrant and Border Vulnerability

Drawing on feminist security studies (Howell and Richter-Montpetit 2019; Shepherd 2008; Wibben 2011; Zalewski 2010) and political geography (Pratt 1999, 2005; Williams 2011; Wright 2006), we develop a discourse-theoretical approach to vulnerability to examine how vulnerable migrants and borders are discursively co-constituted and how these discourses legitimise violent border practices of the EU. We are particularly interested in how both understandings of vulnerability are connected through coloniality, a global power matrix that emerged during colonialism and continues to reproduce hierarchies of gender and race in and through contemporary EU security, border, and migration politics (Bhambra 2017; Bilgiç 2018; De Genova 2018; Kinnvall 2016).

To identify and problematise how discursive constructions of vulnerability function in border security, we follow the assumptions of discourse-theoretical analysis (DTA) which conceptualises discourses as systems of meaning-making (Doty 1996; Shepherd 2008) that are not distinct from material realities (Wright 2006), but co-constitute them. Discourses are dynamic and unstable, linked to adjacent discourses (Doty 1996; Milliken 1999), and structured by, as well as reproductive of power relations. Gender and race as intersecting and co-constitutive categories (Crenshaw 1991) are central to discursive meaning-making. They position subjects and objects in hierarchical relations based on perceived differences between e.g., masculinity/femininity and non-/whiteness that are articulated through coloniality.

As political geographers demonstrate, discourses are also ‘sociospatial circuits’ (Pratt 1999, 218) that ascribe meaning to spaces and produce spatial reality, e.g. of state- and nationhood, as knowable and governable. Feminist geographers (Dixon 2016; Hyndman 2019; Massaro and Williams 2013) particularly emphasise that constructions of spatial reality are intertwined with the construction of gendered and racialised bodies and are therefore always also ‘about control over the marked bodies’ (Dixon and Marston 2011, 446). Sociospatial discourses thus establish a relationship between ‘gendered corporeality and geographies of violence’ (Jackman et al. 2020, 4). They do so particularly through categorising bodies as (un)deserving (Shilliam 2018) of protection and care, and by drawing ‘literal and symbolic boundaries around where state intervention can occur and on whose behalf’ (Williams 2011, 416). Knowledge practices, such as mapping and surveillance, but also assessments of risk and vulnerability, have a central role in this allocation of deservingness. These ‘systems and cultures of scientific domination’ make bodies and spaces known and knowable (Jackman et al. 2020, 3) on the basis of gendered and racialised hierarchies.

Borders are specific spatial constructions that create ‘discursive geographies’ (Dixon and Marston 2011, 446), which strongly circumscribe how spaces and the people that inhabit and move across them are perceived and governed. They are colonial constructs and at the same time crucial to the reproduction of colonial power relations (Mayblin and Turner 2021), because they embody ‘a space whose very existence has been connected with the preservation of colonial and racial global hierarchies’ (Brito 2023, 10). Colonial hierarchies inscribed into borders persist and unfold not only in their exclusionary nature towards racialised bodies from former colonies. They also mark the boundaries of particular value systems and define to which populations these systems apply. In this sense, EU borders enable and legitimise the forceful and violent governing of migrant bodies, and , at the same time, are constructed as bearers of the EU’s self-proclaimed image as a beacon of liberal values – frequently equated with EUropean values – including a universalist understanding of human rights, freedom, and equality in a ‘post-racial’ world (Chakrabarty 2008; Isakjee et al. 2020, 1752). Discursive constructions of EU borders rooted in coloniality therefore enable the bridging of universalist liberal values and the exclusion of and violence against colonised and racialised populations (Walia 2013).

Against this backdrop, we study discourses on vulnerable borders and vulnerable migrants as intertwined and mutually constitutive ‘sociospatial circuits’ (Pratt 1999, 281). We ask how they discursively define (un)deservingness of protection of borders and bodies through which violence is normalised and security actors legitimised (Barkawi and Laffey 2006; Howell and Richter-Montpetit 2019; Wibben 2011). Beyond a critique of gender and race in the stereotypical representations of migrants, e.g., racialised women as particularly vulnerable, we investigate how gendering and racialisation (Machold and Chiniara Charrett 2021; Zalewski 2010) co-constitute borders and migrants as vulnerable. Our intersectional approach entails that we not only look for how vulnerability is associated with femininity and invulnerability with masculinity (Cohn 2014, 53). It also encompasses how these dichotomies are underpinned by racialised Othering and notions of whiteness rooted in coloniality, including constructions of vulnerable territories in need of masculinised protection from racialised migrants, or constructions of the white masculine EUropean Self as the rational and humanitarian protector of vulnerable refugees and territories (Benton 2016; Pallister-Wilkins 2021; Pascucci 2019).

By investigating the relationship between discourses on vulnerable bodies and on vulnerable territories, we grasp how constructions of vulnerability sustain the discriminatory, exclusionary, and violent character of contemporary border regimes and perpetuation of colonial hierarchies. In the following section, we explain our methodological approach to studying the connections, analogies, and reciprocal relations between different gendered and racialised concepts of vulnerability in EU border contexts.

## Methods and Material: Reading Different Meanings of Vulnerability Together

We study the discursive constructions of vulnerability in border security by examining Frontex as a central actor at the EU’s external borders that is institutionally responsible for defining and assessing both the vulnerability of migrants and borders. While EU asylum agencies and international humanitarian organisations, such as EASO and the UNHCR, primarily conduct vulnerability assessments of migrants (Andrijasevic and Walters 2010; Papada 2023), Frontex is gaining relevance as a first contact point between the EU’s border regime and migrants (Frontex 2013c), determining their status on the basis of documents, interviews, and debriefings in camps or at border crossings (Welfens and Bekyol 2021). As foreseen in the New Pact on Migration and Asylum of the European Commission (2020), Frontex’s role in screening migrants for their vulnerability and risky-ness prior to crossing the EU’s external borders is to be further strengthened in order to reduce numbers in border crossings (Standke-Erdmann 2021). With regards to border vulnerability, Frontex oversees assessments that identify and subsequently propose measures to eliminate any eventual weaknesses of borders. The 2016 mandate extension solidified Frontex’s centrality in conducting and supervising systematic vulnerability assessments of individual member states’ borders, defining when and where the agency should become active (Fjørtoft 2020). Both concepts of vulnerability thus become institutionally and politically relevant and linked to each other under the umbrella of Frontex. Considering Frontex’s growing responsibility for identifying vulnerable individuals and groups, and the extended mandate to identify risks and threats to vulnerable borders, it is therefore timely to scrutinise the agency’s conceptualisations of vulnerability as a sense-making device in relation to both assessment practices.

Our material corpus comprises 23 documents published by Frontex between 2013 and 2022 that offer in-depth considerations and definitions of vulnerability and the institutional practices derived from them.^3^ The material can be divided into three categories: 1) training material for border guards, entailing a manual for Fundamental Rights Training for Border Guard’s (Frontex 2013c); 2) risk-analysis reports (RARs) (Frontex 2015, 2016c 2016d, 2017c, 2018b, 2019c, 2020b, 2022) and the related summary booklet of the Common Integrated Risk Analysis Model, CIRAM (Frontex 2013a); and 3) strategy, programming, and governing documents, entailing the Management Board’s decision (Frontex 2016b) to adopt the Common Vulnerability Assessment Methodology (Frontex 2016a), Consolidated Annual Activity Reports (Frontex 2019a, 2020a), Single Programming Reports (Frontex 2016c, 2017b, 2018a, 2019b), a conference report on the Future Developments of Automated Border Control (Frontex 2013b) as well as the Fundamental Rights Strategy (Frontex 2021), and a Special Report by the European Court of Auditors (2021) on Frontex and vulnerability assessments of borders conducted by EU member states.

We understand our material corpus as a ‘structured relational totality’ of texts that are linked to a particular set of social practices (Doty 1996, 6), in this case, the EU’s border regime. We consider the documents as important sites in which meanings are produced in policy-relevant and institutionally consequential ways. By attaining meaning in and through other texts, that is, intertextually (Kristeva 1972; Thibault 1991), they produce meaning through ‘acquir[ing] density … and referential power among themselves’ (Said 2003, 20) with palpable effects within a particular knowledge regime. We therefore analyse the documents as mutually constitutive of one another, producing co-dependent, yet unstable discourses (Doty 1996; Shepherd 2008) on vulnerability.

We draw on Shepherd (2008) in our discourse-theoretical analysis by approaching the material through a double-reading and an identification of rhetorical schemata that emerge around the use of vulnerability. In a first descriptive reading, we examine text passages that relate either to the vulnerability of migrants or borders for definitions and problematisations of vulnerability. We thereby pay particular attention to what kind of subjects and objects are imagined as vulnerable, how, why and who is victimised, as well as who has agency. In a second, theoretically informed reading, we study how gendering (feminisation/masculinisation) and racialisation (Othering, constructions of whiteness) and associated postcolonial hierarchies between (non-)EUropean populations and territories shape and connect constructions of vulnerable bodies and spaces. We present our results by discussing first, how borders and migrants are co-constituted as vulnerable through feminising the object of protection and racialising the alleged causes of vulnerability, and second, how whiteness and masculinity are mobilised through vulnerability discourses to legitimise Frontex – and by extension the EU – as both humanitarian actors and objective producers of neutral knowledge. By establishing a discursive link between the constructions of migrants and borders as vulnerable, we probe into the gendered, racialised, and colonial logics that underpin the marking of bodies and spaces as (un)deserving. Finally, we show how this sustains the EU’s self-ascribed image as a bearer of liberal values, while legitimising the EU’s violent and deadly practices as part of liberal border governance.

## Empirical Analysis

Ensuring the rescue, safety, registration and identification of thousands of vulnerable individuals is an extremely onerous task and one that implies a certain level of inherent risk and vulnerability at the external borders.

(Frontex 2016d, 5)

As the above quote illustrates, vulnerability is ascribed to both migrants and borders in the examined material, sometimes even at the same time. Vulnerability of migrants is a central point of concern that features in all types of documents consulted, but is addressed especially in the risk analysis and training documents. On the one hand, migrant vulnerability is described as characteristic of and embodied by specific groups, most prominently women, children, the elderly, and ill (Frontex 2013c). Yet, multiple ambiguities and a lack of clarity persist as to why certain groups are considered especially vulnerable, and what renders them vulnerable as opposed to other unidentified groups. Limited definition is only provided through categories such as age and gender and/or medical conditions, including illness, general weakness, or pregnancy. Vulnerability of borders as a topic, on the other hand, is relevant within the RARs as well as within strategy and governance documents, and refers to spatial aspects in terms of physical borderlines, member states, regions near borders, and third countries, but also to technologies, infrastructures, procedures, and systems. Vulnerability is also located in national border authorities or embodied through border guards. Per definition, ‘vulnerability assessments are to evaluate the capacity and readiness of each Member State to face challenges at its external borders, including migratory pressure’ and is understood as the capacity of the system to mitigate the threat’ (European Court of Auditors 2021, 11). Here, too, a definition for vulnerability is provided only through other undefined concepts, for instance, ‘factors at the borders or within the EU that might increase or decrease the magnitude or likelihood of the threat’ (Frontex 2016a, 7) or risk understood ‘as a function of threat, vulnerability and impact’ (Frontex 2016a, 6). Through this ambiguity, vulnerability provides a framework through which Frontex discusses a broad array of challenges and problems in border management. Vulnerability is however not only ascribed to both migrants and borders but, as we show in the following, both meanings of vulnerability are discursively connected to each other. Read together, the different but interlinked meanings of vulnerability render the EU border regime and its contested security practices intelligible and acceptable on the basis of a shared colonial matrix that articulates gendered and racialised understandings of (un)deservingness.

### Feminisation of Borders and Racialisation of Threat

In the context of broader humanitarian discourse which is key to sustaining the legitimacy of the EU border regime (Cuttitta 2018; Horsti 2012; Pallister-Wilkins 2015), the notion of vulnerable borders is established across the analysed material. These constructions of borders as vulnerable mirror discourses on vulnerable migrants in that they both follow a narrative of masculinist protectionism (Young 2003) which feminises the vulnerable object of protection and care and constructs it as threatened by a masculinised and racialised Other. This allows for constructing the Self as an ideal masculine protector. When borders, Member States, or the EU are marked as ‘vulnerable’ in the examined documents, they are feminised through association with weakness, defencelessness and permeability opposite ‘flows’ (Frontex 2021, 5; see also Frontex 2019d, 2022) and ‘pressures’ (Frontex 2016a, 22). Their vulnerability emerges through threats from non-European populations and territories and calls for a strong protector, legitimising robust and preventive measures as a reasonable answer to unwanted cross-border movements:

In this context, vulnerability is understood as the factors at the borders or within the EU that might increase or decrease the magnitude or likelihood of the threat. Among the prime factors that affect vulnerability are the geographical attributes of the border areas.

(Frontex 2016a, 7)

The report … featured analyses of key risks affecting the security of the external borders and/or internal security (e.g. smuggling networks in Libya, return system vulnerabilities, and the situation at migrant reception centres).

(Frontex 2017c, 11)

The association of border vulnerability with femininity is sustained by the discourse on migrant vulnerability which establishes and engrains the feminisation of vulnerability by frequently referencing women and children as inherently weak, exploitable, defenceless, and universally prone to ‘sexual violence and exploitation’ (Frontex 2015, 51) and human trafficking by ‘unscrupulous traffickers’ (Frontex 2018b, 37). These groups are thus to be ‘always [prioritised] above all others and are entitled to specific treatment’ (Frontex 2013c, 63). As already shown by others (Freedman 2019; Welfens and Bonjour 2020), such gendered and racialised meanings of vulnerability are expressed particularly in ambiguous understandings of ‘family units’ as self-evidently vulnerable who ‘require […] special attention’ (Frontex 2016d, 18). In our material, this rationale is also evident in essentialist and naturalising gendered assumptions about the nurturing, care-giving, and harmless mother who embodies a natural sense of maternal ‘extraordinary levels of strength and resilience … to sustain the whole family’ (Frontex 2013c, 99).

These feminised constructions of migrant vulnerability and their reliance on gendered discourses on ‘womenandchildren’ (Enloe 2014, 1) as weak, agency-less, and in need of protection provide the grounds for an analogous understanding of vulnerable borders as feminised objects of protection. This analogy emerges from and depends on colonial patterns of racialisation that objectify and dehumanise the colonised feminised subject (Lugones 2010), allowing for an equation of colonised subjects with objects. Constructing borders and bodies as equally vulnerable and inherently brittle, feminised borders and bodies thus share an alleged need for protection. In consequence, the racialised masculinised Other is constructed as always potentially threatening and violent against the feminised object. In Frontex’s assessments of vulnerable borders, the concept of threat is a main variable through which border vulnerability is constructed and allegedly measured. Although none of the documents explicitly detail the markers of (non-)vulnerable borders and territories, considerable space is given to defining third-country territory and subjects as threatening and thus as sources of vulnerability:

Moroccan authorities have also dug a moat and built a high fence on its own territory in the most vulnerable areas of the perimeter near the border with the Spanish cities.

(Frontex 2016d, 21)

The notion of the racialised Other as threat informs and thereby connects both discourses on vulnerability through a colonial dichotomy. More specifically, the idea of vulnerable borders hinges on the dichotomy between vulnerable and non-vulnerable migrants whereby the latter is masculinised and racialised. Frontex, for example, repeatedly links border vulnerability to its broader discursive pattern of associating ‘young single males’ (Frontex 2015, 25; 2016c 2016d, 18) with illegality and crime. This includes the proposition that their false claims of vulnerability (Frontex 2016d, 18) would enhance the vulnerability of ‘truly’ vulnerable women and children:

The crowds also mixed young single men with more vulnerable families, including women and children, sometimes purposely put in front of the groups to facilitate their progression.

(Frontex 2016d, 18)

This racialising discourse is also established in relation to gender-based vulnerabilities of migrants, particularly with regards to human trafficking – a discourse that FitzGerald (2016) has shown to rely heavily on the construction of women as inherently vulnerable in order to legitimise European border regimes. In our analysis, we similarly find that, for example, ‘Nigerian women’ (Frontex 2017a, 15) or individuals from Libya (Frontex 2020b) and Afghanistan (Frontex 2016d) are foregrounded as particularly vulnerable to violence and sexual exploitation, thereby framing gendered violence as originating from and located in formerly colonised spaces that themselves are imagined as vulnerable or dangerous. This is also expressed in racialised notions of the non-Western family (see also Razack 2004), in which women’s and children’s vulnerability is elevated by racialised male family members who are understood as patriarchal and oppressive:

Women migrants should be provided with information directly – there is a risk that information is provided to the male relatives due to cultural assumptions. This may deprive her of vital information when needed and the ability to take independent decisions, and to request protection or assistance.

(Frontex 2013c, 99)

The racialised Other that strongly informs understandings of vulnerable migrants is also constitutive of border vulnerability. This becomes particularly evident in the discussion of ‘push- and pull-factors’ (Frontex 2013a, 9; 2016a, 7), a concept derived from migration studies but often used in contemporary anti-immigration discourse in politics and the media (Richmond 1993). These factors are considered important in assessing vulnerability of borders and contain various racialised assumptions. The list of push-factors includes ‘internal violent conflicts arising from ethnic and/or religious composition of the society, regional conflicts impacting the internal security situation, population under the age of 15’ (Frontex 2016a, 22). Pull-factors that enhance border vulnerability are seen in ‘large communities in Member States’ (Frontex 2013a, 9), ‘perceived easy fraudulent access to international protection and social welfare benefits’, and income disparity/high unemployment between EU and non-EU countries (Frontex 2013a). The factors contributing to border vulnerability intersect with understandings of non-European territories as marked by a constant state of chaos, conflict, instability, and economic underdevelopment that is related to religion and ethnicity, concepts that are common proxies for race (Parashar 2021). They also relate to aforementioned discourses on young, ‘economic’ migrants, imagined as male, as deceiving and exploitative (Kmak 2020; Stachowitsch and Sachseder 2019). By problematising ethnic and religious heterogeneity both as a source of conflict in non-EUropean spaces as well as brought to and encouraged by ‘diaspora communities’ in the EU, ethnonational homogeneity and a nativist view on nationality are positioned as societal ideal and guarantor of stability and peace, associated with and located within EUrope. The proximity between formerly colonised, i.e., dangerous spaces (Doty 1996), to feminised EU borders thereby suggests and gives legitimacy to the need to address heightened levels of vulnerability and risk preventively, e.g. in the form of surveillance through military technologies such as drones (Loukinas 2022), disruptive and deadly border infrastructure (Frowd 2021), or continuous border externalisation (Cobarrubias et al. 2023; Lemberg-Pedersen 2019).

The racialised notion of the Other as threat that makes borders vulnerable also draws on the colonial trope of unknown-ness of both Other vulnerable territories (Bankoff 2001) and of migrant subjects (Sachseder, Stachowitsch, and Binder 2022):

Not knowing who is travelling within the EU is a vulnerability for EU internal security.

(Frontex 2015, 5)

This trope of unknown-ness connects non-EUropean spaces and bodies as threatening through culturalist perceptions of migrants and colonial hierarchies between the knower and the (un)known. This is particularly evident regarding document fraud, a central concern for Frontex, which creates a link between the unknown-ness (and deceptiveness) of migrants and border vulnerability, e.g. in risk analysis that addresses vulnerabilities in detecting ‘fraudulent documents’ (Frontex 2017c, 41). In its discussion of vulnerability assessments of borders, Frontex further widens this concern on a racialised basis to migrants already in the EU who allegedly provide ‘imposter documents that are often recycled by diaspora communities of the same ethnicity’ (Frontex 2013b, 29).

Taken together, the construction of border vulnerability works not only through the feminisation of EU territory in need of masculinised protection but is also enabled through racialised Othering that spatialises threat outside of Europe, embodied in masculinised and racialised ways by young, exploitative, poor, deceptive men, and ethnically and religiously diverse communities outside and within EU borders. Border vulnerability is therefore intersectionally constituted on the basis of colonial hierarchies between femininity/masculinity and non-/whiteness that shape definitions of who or what is (un)deserving of protection and care in the context of liberal border governance.

### Construction of the EU as White and Masculine Protector

The ambiguous and plurivalent use of vulnerability in Frontex’s institutional discourses and practices not only feminises borders as vulnerable objects of humanitarian protection against racialised threats. It also reconstructs the image of Frontex and the wider EU border regime as compatible with liberal values and the logics of liberal governance. By drawing on the authority and legitimacy of masculinity and whiteness, vulnerability discourse strengthens Frontex’s claim to being a humanitarian protector on the one hand, and, on the other, a bearer and producer of neutral knowledge and expertise. In the first instance, the discourse on migrants’ vulnerability is framed as a central humanitarian issue which Frontex is obliged to address in reference to a large body of EU fundamental rights provisions (Frontex 2013c, 2021). This narrative is widened to border vulnerability which is related to the EU’s value-based conduct:

Frontex is a cornerstone of the EU’s area of freedom, security and justice. To help Europe better prepare for future challenges at its external borders, Frontex has begun conducting vulnerability assessments in Member States and already shared its first findings with national authorities.

(Frontex 2018b, 6)

Through linking EU values, humanitarianism, and border vulnerability, Frontex and, by extension, the EU are constructed as the masculine protectors of borders, which are, in turn, feminised as humanitarian objects of care, bolstering normative claims to being a progressive and protective beacon of liberal values. Simultaneously, a technocratic, managerial, and knowledge-based discourse on vulnerability – underpinned by notions of the ‘unknown Other’ - increasingly legitimises the EU as a neutral expert and knowledge producer and sustains claims to objectivity and neutrality (Garlick 2021). In this sense, vulnerability of both borders and migrants is conceptualised as a phenomenon that can be objectively known and neutrally assessed by those with proper tools, training, skills, and knowledge. This idea of neutral knowledge, definability, and measurability of vulnerability in both discourses draws on and reproduces colonial dichotomies between the rational, enlightened EU Self and the emotional, culturally backwards, and irrational Other.

In the case of border vulnerability, the focus is placed on ‘objective indicators’ (Frontex 2016a, 22) for assessments, including technical equipment, systems, resources, infrastructure, and personnel (Frontex 2016a) available to member states to face present and future threats and challenges at external borders (Frontex 2013a). The aim of such assessments is defined as effectiveness, intensification, and standardisation towards a ‘more efficient, high and uniform level of border control’ (Frontex 2016a, 6). Efficiency relates not only to well-managed borders but also to ‘optimal allocation of resources within constraints of budget, staff and … equipment’ (Frontex 2016a, 6). This is made explicit in claims that vulnerability assessments of borders is ‘a technical rather than political exercise’ (Frontex 2016a, 12) and underscored through frequent referrals to data, expertise, and knowledge. This is consistent with previous research that highlighted depoliticisation as a central function of Frontex’s risk analysis and vulnerability assessments (Fjørtoft 2020; Paul 2017) and the ensuing disregard for the simultaneous creation of insecurities for migrants (Perkowski 2021). This depoliticised understanding of border vulnerability is underpinned by processes of Othering that link non-European subjects and territories to crime and illegality and envisions neutral knowledge practices as the solution. This is, for example, evidenced in Frontex’s plans to ‘further develop existing third country intelligence methodology (TCM) to support the implementation of the vulnerability assessment, knowledge on return-related aspects and cross-border crime’ (Frontex 2018a, 35).

The discourse on migrant vulnerability equally establishes scientific and objective factors and expertise as the basis for identifying vulnerability, thereby ingraining neutral knowledge on the Other as key to the allocation of deservingness. For example, training material for border guards stipulates that they are expected to be literate in ‘the culture’, beliefs, or habits of migrants (Frontex 2013c, 63). Knowledge of gender-specific issues represents an important aspect of the border official’s neutral expertise. They are supposed to have knowledge on ‘trafficking in human beings and protection against gender-based persecution’ (Frontex 2021, 10) to objectively and neutrally assess vulnerability claims. Their image is constructed as masculine ‘front-line’ actors and bearers of knowledge with skills to ‘look […] for signs’ of vulnerability (Frontex 2013c, 65). In a responsible and detached manner, they should embody the skills to objectively and neutrally ‘de-escalat[e]’ and ‘polic[e]’ while providing care to the vulnerable ‘Other’ along the lines of EU fundamental rights (Frontex 2013c, 2020b, 49; 2021, 10). They are supposed to identify the need for medical care or potentially criminal behaviour at the ‘brief moment of contact’ (Frontex 2013c, 63) in a not ‘overly sympathetic’ (Frontex 2013c, 98) manner to ensure a professional performance, ‘based on an active and continuing risk analysis’ (Frontex 2013c, 65; 2019c).

The image of the white masculine expert who is knowledgeable about migrants’ culture and their gendered victimisation as well as rational in their assessment co-constitutes the foreign body as ‘emotional’, irrational, steeped in ‘culture’, and potentially dangerous (Frontex 2013c, 65):

The border guard should be prepared for emotional reactions … confronted with persons who may be from countries or cultures where invasive or brutal policing methods are commonly used. After travelling in difficult or even inhumane conditions, the intercepted person may see his or her dream of a better life shattered and may think he or she has nothing to lose anymore. In general, there may be signs of fear, but reactions may also be aggressive or even (self-)destructive. The border guard needs to always aim at de-escalating the situation.

(Frontex 2013c, 65)

As in the above quote, irrational and violent behaviour is explained through migrants’ experiences with ‘brutal police methods’ in non-EUropean ‘countries or cultures’ (Frontex 2013c, 65), obscuring the ways in which migrants experience violence in Europe. While the violence of bordering and the hardship of migrants is at times acknowledged, it is either individualised or projected onto non-EU spaces and ‘cultures’ (Frontex 2018b, 81). The ‘shattering of dreams of a better life’ (Frontex 2013c, 65) that is caused by EU intervention is seen as a necessary consequence, legitimised on the basis of technocratic procedures and neutral assessment. Whilst prioritising the safety of border guards to maintain the functioning of the system, these notions deny those deemed non-vulnerable the admission of insecurities and negate violent experiences people may have had in encounters with EU border authorities.

The threat of unknown-ness connects the discourses on border and migrant vulnerability and posits expertise, quantification, and surveillance – at the heart of managing gendered and racialised subjects and geographies (Hunter 2016; Said 2003; Spivak 1988) – as solutions. This is expressed in demands for measures such as enhanced (automated) screening of migrants, integrated information and data sharing systems, as well as training for border guards and extended rights to use force in potentially threatening situations (Frontex 2016a, 7; 2016a, 22, 2019d, 15).

Increasingly, border-control authorities need to be prepared to manage the flow of vulnerable people, including numerous children. This makes it necessary to focus on further development of specific mechanisms and procedures to meet the needs of this vulnerable group at the EU’s external borders, including all air, land and sea borders.

(Frontex 2018b, 39)

Neutral knowledge is supposed to provide the basis for Frontex’s intervention into migrants’ journeys and legal status as well as into border spaces and even internal territory of Member States and third countries. This is problematic, as colonial Self-Other dichotomies shape the notion of neutral knowledge and related understandings of vulnerability that make it difficult to question and critique violent border practices. Allegedly objective and depoliticised criteria project vulnerabilities and threats endlessly into the future and disregard that borders, as security systems, and bodies, human in their nature, can never be made fully invulnerable (Cohn 2014, 54).

A feminist postcolonial perspective reveals that the interaction between discourses on vulnerability of borders and migrants enables normative claims and reproduces the legitimacy of Frontex and the EU through associations with whiteness and masculinity. This is not only achieved through humanitarianism opposite vulnerable migrants, nor is it established only through neutral, technocratic, and managerial approaches to vulnerable borders. Rather, the ambiguous use of the concept allows for a ‘travelling’ of meanings from one discourse to the other, whereby borders can become objects of humanitarian care, and migrants become objects of ‘systems and cultures of scientific domination’ (Jackman et al. 2020, 3) that completely abstract from human suffering.

## Conclusions

This paper examined different and seemingly unrelated or even contradictory concepts of vulnerability in the EU border security architecture. It argued that studying them together provides insights into the EU’s border politics and selective humanitarian practice and how they legitimise violent border practices as in line with liberal values. By examining the concepts of vulnerability of migrants on the one hand and of borders on the other, we found that vulnerability as a meta-discourse is allocating (un)deservingness to different non-/EUropean spaces and bodies through gendering and racialisation as processes of hierarchisation rooted in coloniality. These discourses are institutionally and politically consequential because they shape how the EU governs cross-border movements and produces, addresses, and challenges related violence and insecurities.

By developing a discourse-theoretical perspective on the connection between border and migrant vulnerability, we have shown how both discourses are co-constituted through gender and race as central meaning-making categories that subsequently enable analogies between bodies and borders. In our material, this is expressed in the feminisation of the object of protection and care, the racialisation of the Other as threat, specifically through the trope of ‘unknown-ness’, and constructions of the EU as a white and masculine security actor, linking to humanitarianism and neutral knowledge production as key legitimising discourses of liberal governance. Thereby, EUrope is constituted as the progressive, emancipated, gender-sensitive and, therefore, superior actor tied to notions of reason, neutrality, fundamental rights, and non-discrimination. Along colonial dichotomies, this is contrasted with non-EUropean contexts which are constructed as emotional, partial, patriarchal, and informed by violent and chaotic ‘culture’. Through the concept of vulnerability, border protection is framed in a way that bridges the seeming contradiction between normative claims to values of care and humanitarianism and the deadly, violent, and repressive character of borders. This works because of the colonial logics underlying liberal values and governance that privilege whiteness and dehumanise the Other (Axster et al. 2021; Isakjee et al. 2020; Mills 2017). Despite continued violence and rising border deaths, Frontex thus emerges as a safeguard of EUropean values and a benevolent masculine protector of both vulnerable migrants and Europe as threatened by the non-EUropean Other.

While the EU’s acknowledgement of harm experienced by (some) migrants during their journeys is therefore important and in principle commendable, our analysis points to how the concept of vulnerability can attain different meanings that ultimately serve the legitimisation of problematic bordering practices. In the case at hand, border vulnerability serves as a central institutional vehicle for rendering measures, such as deterrence, returns, refusals of entry, or pushbacks, intelligible as a legitimate means to protect vulnerable people and territories. At the same time, both uses of vulnerability consistently depoliticise the wider socioeconomic and political causes of migration and invisibilise political decisions and global power relations that lead to both border and migrant vulnerability. As a result, discourses on vulnerable borders and territory silence considerations for migrants’ vulnerability or historical and political contexts which have rendered certain regions and subjects more marginalised and crisis-ridden. But it is also present in references to gender and ‘culture’ as individualised and decontextualised factors determining the vulnerability of migrants. While our study has provided a discourse-theoretical framework and analysis of border vulnerability, further empirical research is needed on how the different concepts of vulnerability relate to, translate into, and manifest in concrete border practices on the ground. This is needed for a better understanding of how security actors give meaning to the multiple and ambivalent uses of vulnerability in documents and how it informs the creation of gendered and racialised hierarchies of deservingness, protection, and care in practice.

Our analysis of different conceptualisations and practices of vulnerability as related to each other finally also sheds light on the growing and increasingly hegemonic role of Frontex in allocating resources, care, and protection in EU migration management. The institutional convergence of concepts of vulnerability within Frontex is particularly concerning in the light of recent and recurring allegations of human rights abuses against the agency. Frontex’s increasing power to act upon its own definitions and assessments of migrants, migration, and borders as risky and/or vulnerable along gendered and racialised lines may give rise and further legitimacy to harmful and violent security practices. Because vulnerability – both as a technocratic and humanitarian category – lends itself well to unchallenged institutional claims to power and resources, its increasing centrality could paradoxically contribute to relieving Frontex of responsibility for exacerbating migrants’ vulnerability.

## Notes

1. IBM assembles various practices ranging from border control, Search and Rescue, returns, risk analysis, research and development, agreements with third countries, monitoring of migratory flows, and prevention and detection of cross-border crime (Frontex 2019d). With the two mandate extensions of Frontex in 2016 and 2019, IBM has been put largely under the authority of Frontex, making the agency an increasingly powerful actor (Perkowski 2021).

2. We apply the term ‘EUropean’ (Mamadouh and Bialasiewicz 2016, 129) to elucidate the relation between the EU as an institutional entity and the normative notions that underlie the idea of Europe.

3. While the examined documents give some insights into the way vulnerability assessments are supposed to be conducted, in-depth fieldwork would be needed to understand the concrete practices involved in deeming borders as vulnerable based on surveillance and other data. For vulnerability assessments of migrants, several scholars (Bird 2022; Papada 2023; Sözer 2019; Spathopoulou, Carastathis, and Tsilimpounidi 2022) have already started this important work. Our analysis however focuses on the discursive constructions of vulnerability and the associated meaning-making processes observable in the documents. This gives insights into how EU institutions imagine the causes and consequences of vulnerability, how they justify the allocation of deservingness on this basis, and how this relates to the legitimisation of their practices, not necessarily into the actual practices during risk assessments.
